# Prevalence of the Hippo Effectors YAP1/TAZ in Tumors of Soft Tissue and Bone

**DOI:** 10.1038/s41598-019-56247-8

**Published:** 2019-12-23

**Authors:** Ilka Isfort, Sandra Elges, Magdalene Cyra, Ruth Berthold, Marcus Renner, Gunhild Mechtersheimer, Pierre Åman, Olle Larsson, Nancy Ratner, Susanne Hafner, Thomas Simmet, Christoph Schliemann, Claudia Rossig, Uta Dirksen, Inga Grünewald, Eva Wardelmann, Sebastian Huss, Wolfgang Hartmann, Marcel Trautmann

**Affiliations:** 1 Division of Translational Pathology, Gerhard-Domagk-Institute of Pathology, Münster University Hospital, Münster, Germany; 2 Gerhard-Domagk-Institute of Pathology, Münster University Hospital, Münster, Germany; 3 Institute of Pathology, Heidelberg University Hospital, Heidelberg, Germany; 4 Sahlgrenska Cancer Center, Department of Pathology and Genetics, Institute of Biomedicine, Sahlgrenska Academy at University of Gothenburg, Gothenburg, Sweden; 5 Departments of Oncology & Pathology, The Karolinska Institute, Stockholm, Sweden; 6 Division of Experimental Hematology and Cancer Biology, Cincinnati Children’s Hospital Medical Center, Cincinnati, United States of America; 7 Institute of Pharmacology of Natural Products & Clinical Pharmacology, Ulm University, Ulm, Germany; 8 Department of Medicine A, Hematology and Oncology, Münster University Hospital, Münster, Germany; 9 Department of Pediatric Hematology and Oncology, University Children´s Hospital Münster, Münster, Germany; 10 Cells in Motion Cluster of Excellence (EXC 1003 – CiM), University of Münster, Münster, Germany; 11 Pediatrics III, West German Cancer Center, University Hospital Essen, Essen, Germany; 12 German Cancer Consortium (DKTK), Essen, Germany

**Keywords:** Predictive markers, Targeted therapies, Sarcoma, Tumour biomarkers, Oncogenesis

## Abstract

Tumors of soft tissue and bone represent a heterogeneous group of neoplasias characterized by a wide variety of genetic aberrations. Albeit knowledge on tumorigenesis in mesenchymal tumors is continuously increasing, specific insights on altered signaling pathways as a basis for molecularly targeted therapeutic strategies are still sparse. The aim of this study was to determine the involvement of YAP1/TAZ-mediated signals in tumors of soft tissue and bone. Expression levels of YAP1 and TAZ were analyzed by immunohistochemistry in a large cohort of 486 tumor specimens, comprising angiosarcomas (AS), Ewing sarcomas, leiomyosarcomas, malignant peripheral nerve sheath tumors (MPNST), solitary fibrous tumors, synovial sarcomas (SySa), well-differentiated/dedifferentiated/pleomorphic and myxoid liposarcomas (MLS). Moderate to strong nuclear staining of YAP1 and TAZ was detected in 53% and 33%, respectively. YAP1 nuclear expression was most prevalent in MPNST, SySa and MLS, whereas nuclear TAZ was predominately detected in AS, MLS and MPNST. In a set of sarcoma cell lines, immunoblotting confirmed nuclear localization of YAP1 and TAZ, corresponding to their transcriptionally active pool. Suppression of YAP1/TAZ-TEAD mediated transcriptional activity significantly impaired sarcoma cell viability *in vitro* and *in vivo*. Our findings identify nuclear YAP1 and TAZ positivity as a common feature in subsets of sarcomas of soft tissue and bone and provide evidence of YAP1/TAZ-TEAD signaling as a specific liability to be considered as a new target for therapeutic intervention. Nuclear YAP1/TAZ expression may represent a biomarker suited to identify patients that could benefit from YAP1/TAZ-TEAD directed therapeutic approaches within future clinical trials.

## Introduction

The Yes associated protein 1 (YAP1) and transcriptional co-activator with PDZ-binding motif (TAZ) are central effectors of the Hippo signaling pathway, which is essential in regulating tissue homeostasis and organ size^1^. The signaling cascade’s key components include mammalian STE20-like protein kinase 1/2 (MST1 and MST2) and protein salvador homolog 1 (SAV1), which in complex phosphorylate the large tumor suppressor kinase 1/2 (LATS1 and LATS2) and Mps one binder kinase activator 1 (MOB1), a LATS1/2 adaptor. Activated LATS1/2 in turn phosphorylates YAP1 and TAZ, leading to their cytoplasmic retention through the interaction with a member of the 14-3-3 protein family and/or ubiquitin-mediated degradation^2,3^. In the inactive state of the Hippo signaling cascade, non-phosphorylated YAP1 and TAZ translocate into the nucleus and interact with transcription factors such as TEA domain transcription factor 1-4 (TEAD 1-4) to induce expression of downstream target genes that promote e.g. cell proliferation and suppression of apoptosis. The essential role of YAP1 and TAZ in different epithelial malignancies is well established^4^. However, evidence on a functional role of YAP1 and TAZ in mesenchymal tumorigenesis is sparse and warrants further investigation^5–8^. Transgenic mice harboring gene mutations in key modulator or effector proteins of the Hippo signaling pathway were found to develop different types of sarcoma^9,10^.

In this study, we analyzed a large cohort of specimens of soft tissue and bone tumors and sarcoma cell lines to delineate the prevalence of YAP1/TAZ expression in order to identify subgroups of tumors which might be particularly dependent on Hippo-YAP1/TAZ signals and therefore might profit from therapeutic YAP1/TAZ inhibition.

## Materials and Methods

### Tumor specimens and tissue microarrays (TMA)

Tissue microarrays were constructed from 486 formalin-fixed, paraffin-embedded tumor specimens of soft tissue and bone, selected from the archives of the Gerhard-Domagk-Institute of Pathology (Münster University Hospital, Germany) and the Department of Pathology in Heidelberg. Diagnoses were reviewed by at least three experienced pathologists (SH, GM, EW, WH) based on current World Health Organization criteria^11^. FISH and/or RT-PCR analyses were performed to detect the pathognomonic chromosomal aberrations, confirming the diagnosis of (a) Ewing sarcoma (*EWSR1-FLI1* or *EWSR1-ERG*), (b) solitary fibrous tumor (*NAB2-STAT6*), (c) synovial sarcoma (*SS18-SSX1/2*), (d) de/well-differentiated liposarcoma (*MDM2* and/or *CDK4* gene amplification), and (e) myxoid liposarcoma (*FUS-DDIT3*). In total, a large cohort of 10 tumor entities of soft tissue and bone was analyzed, comprising: angiosarcomas (AS; n = 29), Ewing sarcomas (EwS; n = 20), leiomyosarcomas (LMS; n = 68), malignant peripheral nerve sheath tumors (MPNST; n = 45), solitary fibrous tumors (SFT; n = 36), synovial sarcomas (SySa; n = 65), well-differentiated liposarcomas (WDLS; n = 55), dedifferentiated liposarcomas (DDLS; n = 74), myxoid liposarcomas (MLS; n = 85), and pleomorphic liposarcomas (PLS; n = 9). From each individual paraffin block, two representative areas were selected to account for potential heterogeneity, e.g. with regard to the round cell content of MLS. Occasionally occurring necrobiotic areas were excluded from TMA sampling. Scientific analysis of the cohort of mesenchymal tumors was approved by the Ethics Review Board of the University of Münster (2015-548-f-S) and the Medical Faculty Heidelberg (206/2005 and 207/2005). Experiments were conformed to the principles set out in the World Medical Association Declaration of Helsinki and the United States Department of Health and Human Services Belmont Report.

### Immunohistochemistry (IHC)

Immunohistochemistry was performed with a BenchMark ULTRA Autostainer (VENTANA/Roche) on 3 μm TMA sections. The staining procedure included heat-induced epitope retrieval using Tris-Borate-EDTA buffer (pH 8.4; 95–100 °C, 32–72 min), incubation with primary antibodies for 44 min (YAP) or 32 min (TAZ), and signal detection using the OptiView DAB IHC Detection Kit (VENTANA/Roche). Following primary antibodies were used: YAP (monoclonal rabbit, D8H1X, 1:100, catalog no. 14074, Cell Signaling), and TAZ (polyclonal rabbit, 1:150, catalog no. HPA007415, Sigma Aldrich). Nuclear immunoreactivity was assessed using a semiquantitative score (0, negative; 1, weak; 2, moderate; and 3, strong) defining the staining intensity in the positive control (hepatocellular carcinoma) as strong. Negative control stainings using an appropriate IgG subtype (DCS) were included. Microscopy of the immunostains included an initial pre-screen at low power (4x) to identify regions with a technically optimal staining result. Subsequently, detailed analysis of at least 20x power fields and, if suitable, high power fields (40x) was performed to evaluate the stainings according to routine algorithms employed e.g. in neuroendocrine tumor diagnostics. Only tumors with at least moderate staining (semiquantitative score ≥2) and ≥30% positive cells were considered as positive for the purposes of the study. The IHC readers were blinded to outcome data, the score threshold (positive = semiquantitative score ≥2) was pre-specified without prior analyses of the clinical course.

### Immunoblotting

Subcellular protein fractionation was performed using the NE-PER Nuclear and Cytoplasmic Extraction Reagents kit (Thermo Fisher Scientific). Following primary antibodies were used: β-actin (monoclonal mouse, AC-15, 1:10.000, catalog no. A5441, Sigma-Aldrich), CTGF (polyclonal rabbit, 1:1.000, catalog no. 6992, Abcam), FOXM1 (polyclonal rabbit, K-19,1:500, catalog no. sc-500, Santa Cruz Biotechnology), GAPDH (monoclonal rabbit, D16H11, 1:1.000, catalog no. 5174, Cell Signaling Technology), Histone H3 (monoclonal rabbit, D1H2, 1:1.000, catalog no. 4499, Cell Signaling Technology), PLK1 (monoclonal rabbit, 208G4, 1:1.000, catalog no. 4513, Cell Signaling Technology), YAP (monoclonal rabbit, D8H1X, 1:1.000, catalog no. 14074, Cell Signaling Technology), and YAP/TAZ (monoclonal rabbit, D24E4, 1:1.000, catalog no. 8418, Cell Signaling Technology). Secondary antibody labeling as well as immunoblot development was performed using an enhanced chemiluminescence detection kit (SignalFire ECL Reagent; Cell Signaling Technology) and the Molecular Imager ChemiDoc System (Image Lab Software; Bio-Rad Laboratories).

### Cell culture

The human MLS1765-92 myxoid liposarcoma (CVCL_S817; expressing *FUS-DDIT3;* contributed by Pierre Åman)^12^, CME-1 synovial sarcoma (CVCL_N586; monophasic; expressing *SS18-SSX2;* contributed by Olle Larsson)^13^, ST88-14 malignant peripheral nerve sheath tumor (CVCL_8916; contributed by Nancy Ratner)^14^ and TC-32 Ewing sarcoma (CVCL_7151; expressing *EWSR1-FLI1*; received from the Children’s Hospital Los Angeles) cell lines were cultured in Roswell Park Memorial Institute medium 1640 (RPMI; MLS1765-92, CME-1 and TC-32) or Dulbecco’s Modified Eagle medium (DMEM; ST88-14) supplemented with 10% fetal bovine serum (FBS; Life Technologies). Cell line identity was verified utilizing the Cell Authentification SNP Profiling service by Multiplexion and *FUS-DDIT3*, *SS18-SSX*2 or *EWSR1-FLI1* gene fusion specific RT-PCR. Cells were grown under standard incubation conditions (37 °C, humidified atmosphere, 5% CO_2_) and mycoplasma testing was performed quarterly by standardized PCR. Cells were passaged for a maximum of 20 to 30 culturing cycles between thawing and use in the described experiments. To study the effects of increasing concentrations (0.25–1.0 µmol/L) of verteporfin^15–19^, CME-1 cells were grown in medium supplemented with 2% FBS. Cell lysis, protein extraction and immunoblotting were performed 16 h after treatment as previously described^20^.

### Cell viability assay

To determine the effects of YAP1/TAZ signaling suppression by inhibition of the YAP1/TAZ-TEAD transcription complex, MLS1765-92 (1.5 × 10^3^), CME-1 (6 × 10^3^), and ST88-14 (2.5 × 10^3^) cells were seeded in 96-well cell culture plates (100 µl of medium supplemented with 2% FBS) and exposed to increasing concentrations of verteporfin (0.125–2 µmol/L) for 72 h. Cell viability was measured using the Cell Proliferation Kit I (MTT) (Roche) as previously described^21^. Verteporfin (C_41_H_42_N_4_O_8_; CAS#: 129497-78-5; Targetmol)^15–19^ was dissolved in dimethyl sulfoxide (DMSO; Sigma-Aldrich). The final DMSO concentration did not exceed 0.2% (v/v) and an appropriate DMSO vehicle control was included for all *in vitro* and *in vivo* applications. At least three independent experiments were performed (each in quintuplicates) and results were calculated as mean + SEM.

### Luciferase assay

To assess the ability of verteporfin to suppress YAP1/TAZ-TEAD complex formation and associated transcriptional activity, CME-1 cells were transfected with 8xGTIIC TEAD luciferase reporter plasmid DNA (Addgene #34615)^22^. After 5 h, transfection medium was replaced with medium containing 0.075–0.15 µmol/L verteporfin and supplemented with 2% FBS. After incubation for 48 h, cells were lysed and luciferase activity was measured in triplicates using the Dual-Luciferase reporter assay system (Promega) as described previously^13^. Firefly luciferase activity was normalized to the co-transfected Renilla pRL-TK control plasmid (Promega) to account for potential differences in transfection efficiency.

### RNA interference (RNAi)

To exclude unspecific off target effects, a set of pre-validated Stealth siRNAs for *YAP1* (Set of 3): #1 = HSS115942, #2 = HSS115944, #3 = HSS173621, TAZ (*WWTR1*) (Set of 3): #1 = HSS119545, #2 = HSS119546, #3 = HSS119547 and a non-targeting negative control siRNA (BLOCK iT Alexa Fluor Red Fluorescent Control; all purchased by Life Technologies) were applied. CME-1 cells were cultured in 75 cm^2^ culture flasks (medium supplemented with 2% FBS) and transfected with the indicated siRNA using Lipofectamine RNAiMAX (Life Technologies) 72 h prior to MTT cell viability assays or protein lysis for immunoblotting to verify knockdown efficiency.

### *In vivo* efficiency of verteporfin in cell line based chick embryo chorioallantoic membrane (CAM) studies

For *in vivo* confirmation, we used the chick embryo chorioallantoic membrane (CAM) *in vivo* model as previously reported and validated for anticancer agents^21,23–25^. Due to the presence of vascular supply and the absence of an immune response from the graft, the CAM enables the transplantation of human cancer cells and the subsequent development of solid tumor xenografts in a three-dimensional *in vivo* microenvironment. The CAM model matches the 3 R recommendations to reduce mammalian animal experiments and is regarded as reproducible, reliable, and effective^26^. Seven days after fertilization, CME-1 cells (1.5 × 10^6^ cells/egg; dissolved in medium/Matrigel 1:1, v/v) were xenografted onto the chick embryo CAM and incubated with 60% relative humidity at 37 °C. Topical treatment with verteporfin (1 µmol/L) or DMSO vehicle control (0.2% DMSO in NaCl 0.9%) was initiated on day 8 and recapitulated for two consecutive days. Three days after treatment initiation, CAM xenografts were imaged, explanted, and fixed (5% PFA). Tumor volume (TV, mm^3^) was calculated according to the formula: TV = length (mm) × width² (mm) × π/6. All *in vivo* studies were performed in accordance with the standards of the National and European Union guidelines.

### Statistical analysis

Statistical analysis was performed using paired or unpaired two-tailed t-test or Fisher exact test as appropriate. Statistical differences were considered significant at P < 0.05 (*), P < 0.01 (**) and P < 0.001 (***). Computations were performed using GraphPad Prism (GraphPad Software).

## Results

### Nuclear expression of YAP1 and TAZ in patient tumor samples of soft tissue and bone

**Figure 1:**
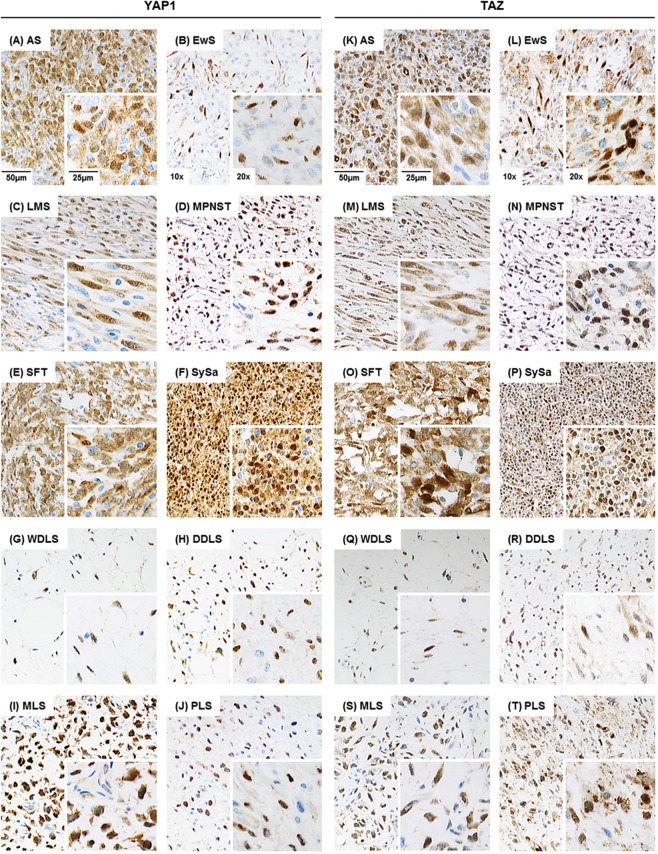
YAP1 and TAZ immunohistochemistry in soft tissue and bone tumor specimens (n = 486). Immunohistochemical staining shows strong nuclear expression of YAP1 (**A**–**J**; left panel) and TAZ (**K**–**T**; right panel) in representative cases of angiosarcomas (AS; n = 29), Ewing sarcomas (EwS; n = 20), leiomyosarcomas (LMS; n = 68), malignant peripheral nerve sheath tumors (MPNST; n = 45), solitary fibrous tumors (SFT; n = 36), synovial sarcomas (SySa; n = 65), well-differentiated liposarcomas (WDLS; n = 55), dedifferentiated liposarcomas (DDLS; n = 74), myxoid liposarcomas (MLS; n = 85), and pleomorphic liposarcomas (PLS; n = 9) (original magnification: x10, inset x20).

**Figure 2:**
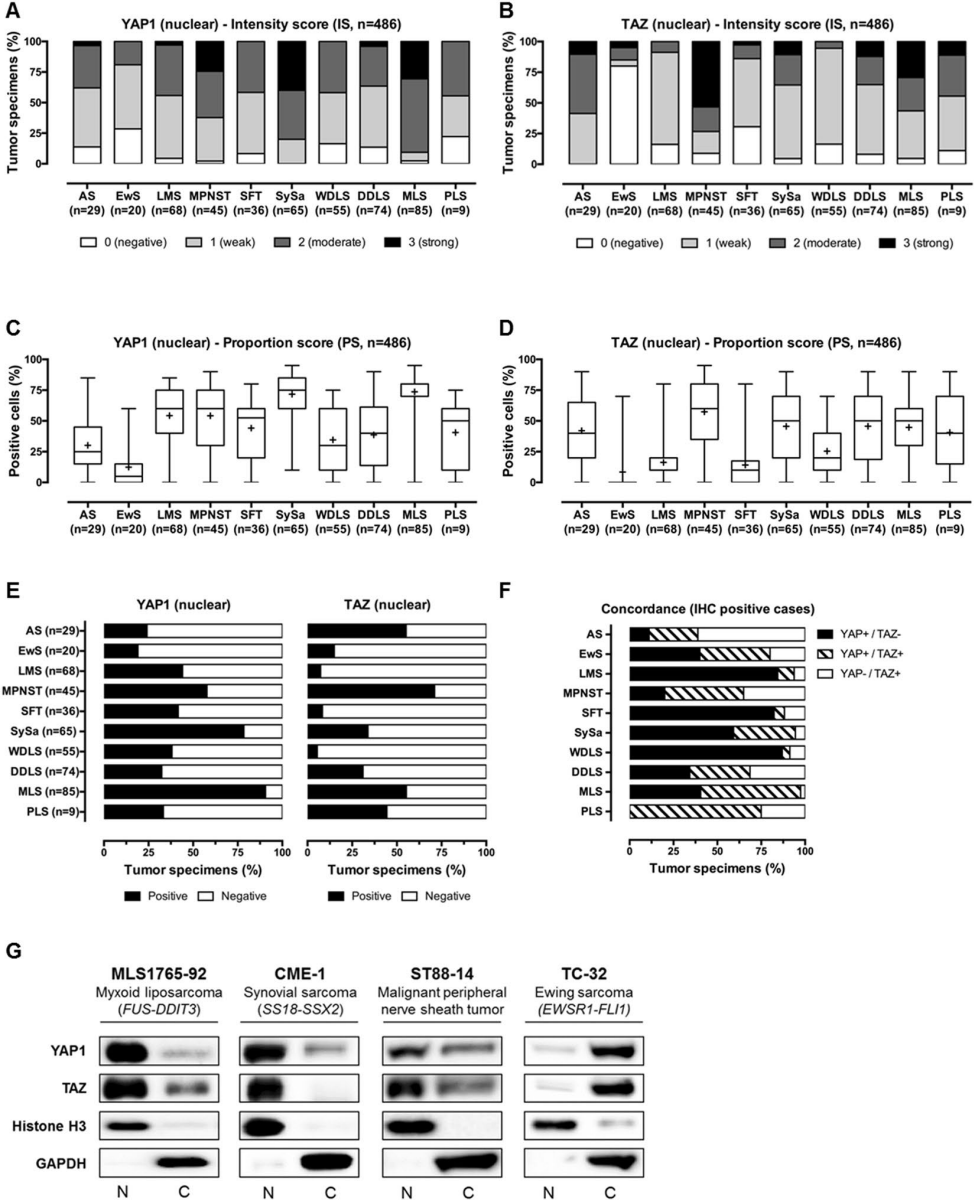
Systematic overview and functional implications of nuclear YAP1/TAZ levels in tumor specimens of soft tissue and bone. (**A**,**B**) Immunohistochemical spectrum of tumor tissue specimens summarized as bar charts (intensity score) and (**C**,**D**) box plots (proportion score; shown are whiskers from minimum to maximum, 25th percentile, median and 75th percentile; +represents the mean of positive cells) for nuclear YAP1 and TAZ. Nuclear immunoreactivity was assessed using a semiquantitative score (0, negative; 1, weak; 2, moderate; and 3, strong) defining the staining intensity in the positive control (hepatocellular carcinoma) as strong. (**E**) Overall immunopositivity for nuclear YAP1 and TAZ. Only tumors with at least moderate staining (semiquantitative score ≥2) and ≥30% positive cells were considered positive for the purposes of the study. (**F)** Concordance of nuclear YAP1 and nuclear TAZ IHC-positivity. (**G**) Nuclear expression of YAP1 and TAZ in sarcoma cell lines. Immunoblotting demonstrates separated nuclear (N) and cytoplasmic (C) YAP1 and TAZ protein fractions in MLS1765-92 myxoid liposarcoma, CME-1 synovial sarcoma, ST88-14 malignant peripheral nerve sheath tumor and TC-32 Ewing sarcoma cells. Nuclear localization of YAP1/TAZ serves as indicator of YAP1- and TAZ-mediated transcriptional activity. Histone H3 and GAPDH were used as controls for the nuclear and cytoplasmic fraction, respectively. One of at least three independent experiments with comparable results is shown.

To determine the involvement of YAP1/TAZ-mediated signal transduction, expression levels and nuclear localization (corresponding to the transcriptionally active pool of YAP1 and TAZ) were examined in a large cohort of 486 tumors of soft tissue and bone using immunohistochemistry (Figs. 1 and 2). Tissue specimens were analyzed with regard to nuclear staining intensity (IS; Fig. 2A,B) and proportion of cells (PS; Fig. 2C,D) demonstrating nuclear YAP1/TAZ immunoreactivity. Overall, moderate to strong nuclear staining of YAP1 and TAZ was detected in 53% (258/486) and 33% (158/486) of soft tissue and bone tumor specimens, respectively (Fig. 2E and summarized in Table 1). 47% (228/486; YAP1) and 67% (328/486; TAZ) of the tissue specimens displayed < 30% immunoreactive cells and/or only weak nuclear YAP1/TAZ staining intensity, and thus were considered as negative. Nuclear YAP1 IHC-positivity was most prevalently detected in MPNST (58%; 26/45), SySa (78%; 51/65) and MLS (91%; 77/85) samples. Moderate to strong nuclear staining of YAP1 was detected in less than half of AS (24%; 7/29), EwS (20%; 4/20), LMS (44%; 30/68), SFT (42%; 15/36), WDLS (38%; 21/55), DDLS (32%; 24/74) and PLS (33%; 3/9) tissue specimens (Figs. 1 and 2A,C,E). Nuclear TAZ immunoreactivity was demonstrated in a variety of sarcoma types, most significantly in AS (55%; 16/29), MPNST (71%; 32/45) and MLS (55%; 47/85). In SySa, nuclear TAZ IHC-positivity was detected in 22 out of 65 (34%) tissue specimens, while 31% (23/74) of DDLS and 44% (4/9) of PLS were positive. Only very occasionally, tissue specimens of LMS (7%; 5/68), SFT (8%; 3/36), WDLS (5%; 3/55) and EwS (15%; 3/20) showed nuclear TAZ staining (Figs. 1 and 2B,D,E). Concordance of nuclear YAP1 and TAZ immunoreactivity was demonstrated in a broad range of soft tissue and bone tumor specimens (4–75%). YAP1 and TAZ nuclear staining was simultaneously detected in more than 35% of IHC-positive EwS (40%), MPNST (45%), SySa (35%), MLS (57%) and PLS (75%) tissue specimens. Overall, YAP1 was shown to be the predominantly “activated” Hippo signaling effector in LMS, SFT, SySa, WDLS and MLS. In contrast, nuclear TAZ expression was predominant in AS and MPNST (Fig. 2F).

**Table 1:** Summary of the immunohistochemical analysis of nuclear YAP1 and TAZ expression in tumors of soft tissue and bone.

Type	n	YAP1	TAZ
Angiosarcoma (AS)	29	7 (24%)	16 (55%)
Ewing sarcoma (EwS)	20	4 (20%)	3 (15%)
Leiomyosarcoma (LMS)	68	30 (44%)	5 (7%)
Malignant peripheral nerve sheath tumor (MPNST)	45	26 (58%)	32 (71%)
Solitary fibrous tumor (SFT)	36	15 (42%)	3 (8%)
Synovial sarcoma (SySa)	65	51 (78%)	22 (34%)
Well-differentiated liposarcoma (WDLS)	55	21 (38%)	3 (5%)
Dedifferentiated liposarcoma (DDLS)	74	24 (32%)	23 (31%)
Myxoid liposarcoma (MLS)	85	77 (91%)	47 (55%)
Pleomorphic liposarcoma (PLS)	9	3 (33%)	4 (44%)
**Total**	**486**	**258** (**53%)**	**158** (**33%)**

Only tumors with at least moderate staining (semiquantitative score ≥2) and ≥30% positive cells were considered positive for the purposes of this study.

### Nuclear expression of YAP1 and TAZ in sarcoma cell lines

To further explore the involvement of YAP1 and TAZ signaling in sarcomagenesis, we examined nuclear YAP1 and TAZ protein levels (corresponding to the transcriptionally active pool) in a panel of MLS (MLS1765-92; *FUS-DDIT*3 translocated), SySa (CME-1; *SS18-SSX2* translocated), MPNST (ST88-14) and EwS (TC-32; *EWSR1-FLI1* translocated) cell lines using immunoblotting. In accordance to the demonstrated strong expression levels and nuclear YAP1/TAZ immunoreactivity in primary tumor tissue specimens, fractionation experiments demonstrated that YAP1 and TAZ were predominantly localized in the nucleus of MLS, SySa and MPNST cells (Fig. 2G), whereas YAP1 and TAZ were predominantly localized in the cytoplasm of TC-32 EwS cells (comparable to the low YAP1/TAZ nuclear expression detected by IHC), providing evidence that YAP1/TAZ signaling might represent a unifying feature in MLS, SySa and MPNST.

### Sensitivity of sarcoma cells to inhibition of YAP1/TAZ-TEAD activity *in vitro*

**Figure 3:**
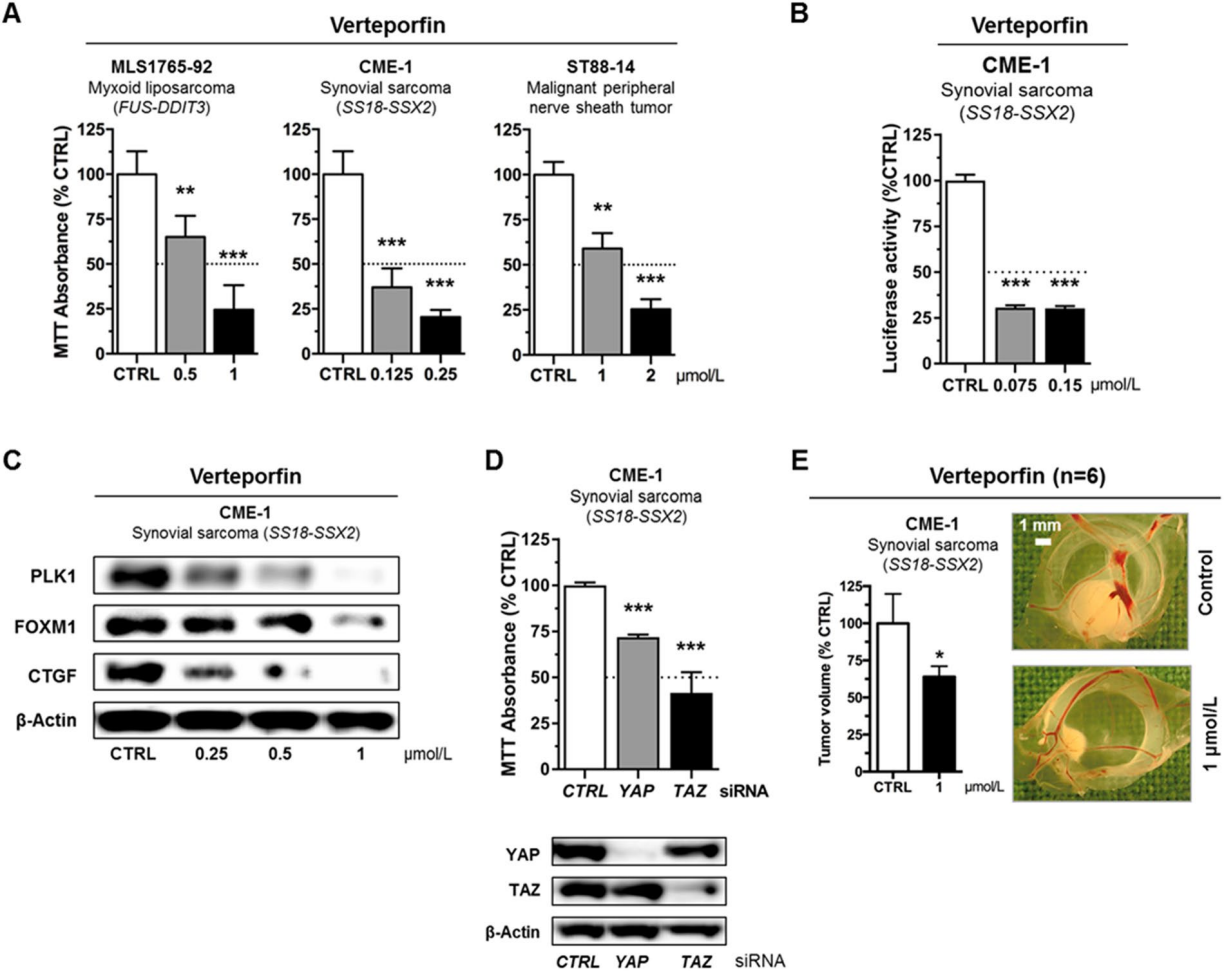
Requirement for YAP1/TAZ activity and sensitivity of sarcoma cell lines to pharmacologic YAP1/TAZ-TEAD inhibition. (**A**) Viability of sarcoma cells was significantly reduced by treatment with increasing concentrations of the YAP1/TAZ-TEAD inhibitor verteporfin (0.125–2 µmol/L; ***P < 0.001; **P < 0.01) *in vitro*. At least three independent experiments were performed; results are represented as mean + SEM of one representative experiment performed in quintuplicates. (**B**) YAP1/TAZ-responsive luciferase reporter activity in CME-1 cells treated with 0.075–0.15 µmol/L verteporfin. Relative luciferase activity is displayed normalized to the DMSO vehicle control. Bars and error bars represent the mean + SD of one representative experiment performed in quintuplicates. (**C**) Reduced expression levels of YAP1/TAZ-TEAD downstream targets PLK1, FOXM1 and CTGF in CME-1 cells treated with 0.25–1 µmol/L verteporfin for 16 h. One of at least three independent experiments with similar results is shown. (**D**) Significantly inhibited cell viability of CME-1 cells following RNAi-mediated *YAP1* (siRNA#3) and TAZ (*WWTR1*; siRNA#3) knockdown. Bars and error bars represent the mean + SD. The blots represent one of at least three independent experiments with similar results. (**E**) Significantly suppressed tumor growth of CME-1 cells on chick embryo CAM following treatment with 1 µmol/L verteporfin *in vivo*. Shown are tumor volumes and representative photographs of CAM xenografts. Bars and error bars represent the mean + SEM of six xenografts.

Our immunohistochemical analyses suggest that aberrant YAP1/TAZ activity might represent a therapeutic target in MLS, SySa and MPNST. We therefore evaluated the growth and viability of sarcoma cells in the presence of verteporfin (0.125–2 µmol/L), an FDA-approved second-generation photosensitizer for the treatment of age-related macular degeneration that inhibits YAP1/TAZ signaling by suppression of the YAP1/TAZ-TEAD transcription complex and augmenting YAP1 sequestration in the cytoplasm^15–19^. MTT assays showed that verteporfin significantly suppressed the viability of all three sarcoma cell lines in a dose-dependent manner (Fig. 3A; ***P < 0.001, **P < 0.01). In CME-1 cells, the inhibitory effects of verteporfin could be attributed to significantly decreased YAP1/TAZ-TEAD luciferase reporter activity (Fig. 3B) and were accompanied by dose-dependent changes in the expression of the YAP1/TAZ-TEAD downstream targets PLK1, FOXM1 and CTGF (Fig. 3C). To confirm the indicated requirement for YAP1/TAZ in sarcomagenesis, we transiently suppressed *YAP1* and *TAZ* expression in CME-1 cells. RNAi-mediated depletion of endogenous *YAP1* and *TAZ* inhibited the viability of CME-1 cells (Fig. 3D), indicating that SS18-SSX-positive human CME-1 cells are dependent on YAP1/TAZ signaling. Collectively, these results imply sensitivity of MLS, SySa and MPNST to pharmacologic inhibition of nuclear YAP1/TAZ-TEAD transcriptional activity *in vitro*, supporting the idea that dependency on YAP1/TAZ-TEAD signaling may provide a new target for therapeutic intervention in sarcoma patients with nuclear YAP1/TAZ activity.

### *In vivo* efficacy of YAP1/TAZ inhibition against sarcoma xenografts

To verify the effect of YAP1/TAZ inhibition on sarcoma cell growth *in vivo*, we deposited CME-1 cells on the chick embryo CAM. Topical administration of verteporfin (1 µmol/L) to established CME-1 xenografts (n = 6) resulted in a significant reduction of tumor volume compared to the DMSO vehicle-treated control group (Fig. 3E; *P < 0.05), further supporting the idea that aberrant YAP1/TAZ signaling could represent a new target for therapeutic intervention in sarcoma patients.

## Discussion

Tumors of soft tissue and bone represent a heterogeneous group of neoplasias characterized by a variety of genetic alterations. Due to limited therapeutic options, identification of novel molecular targets is of particular importance in these rare malignancies. Since inhibition of chimeric fusion proteins (driving tumorigenesis in a subset of sarcomas) represents a therapeutic challenge, it appears most promising to interfere with aberrantly activated signaling pathways. Recent studies demonstrate an essential role of YAP1/TAZ in sarcomagenesis implying that a YAP1/TAZ-directed therapeutic approach could represent a rational strategy in selected entities^5–10,27–31^. The aim of this study was an unbiased immunohistochemical approach to identify additional subgroups of tumors of soft tissue and bone with elevated YAP1/TAZ nuclear expression levels, which thus might be suitable for YAP1/TAZ inhibitory approaches.

Our observations in a large cohort of tumors (n = 486) comprising 10 different sarcoma subtypes document highest YAP1 and/or TAZ nuclear levels in myxoid liposarcomas (MLS), synovial sarcomas (SySa), malignant peripheral nerve sheath tumors (MPNST) and angiosarcomas (AS), implying a pathogenic role of YAP1/TAZ activity. Overall, nuclear YAP1 and/or TAZ IHC-positivity was observed in about 53% and 33% of all analyzed tumors of soft tissue and bone, respectively. Intratumoral concordance of nuclear YAP1 and TAZ positivity was demonstrated in more than 28% of all SySa, pleomorphic liposarcoma (PLS), MPNST and MLS tissue specimens, though a considerable number of tumors appeared to express only one of the two oncoproteins. For instance, WDLS, SFT and LMS were almost exclusively positive for nuclear YAP1, whereas higher nuclear TAZ levels were detected in MPNST and AS tumor specimens. In contrast, Fullenkamp *et al*., analyzing only a smaller cohort (n = 159) with less than 10 samples for the majority of all investigated sarcoma subtypes, reported nuclear TAZ positivity in up to 2/3 of all cases^5^. Our immunohistochemical findings are in line with a previous study reported by Tsuneki *et al*. providing evidence that Hippo pathway inactivation represents an essential feature of AS^32^.

As the majority of the investigated samples were derived from our consult files, information on the clinical course of the patients (e.g. relapses, distant metastasis) was sparse. A consistent correlation of YAP1/TAZ expression with clinical parameters including tumor progression was not feasible in this cohort. Given the molecular heterogeneity of sarcomas as a group with a majority of tumors showing complex karyotypes and other entities being characterized by defined chromosomal translocations against the background of a largely stable genome it is intuitive that the biological (and clinical) role of YAP1/TAZ may well be very much different in various entities. To further elucidate if there is a pattern of mutual exclusiveness of YAP1 and TAZ in subgroups, we evaluated data from The Cancer Genome Atlas (TCGA). At the time of evaluation (Nov 2019), the grouped soft tissue/sarcoma (SARC) gene expression data set (HTSeq, Illumina) comprised 265 samples (combining various histological sarcoma types). The available data did not reveal a significant correlation between (i) *YAP1* or TAZ (*WWTR1)* expression or (ii) YAP1/TAZ expression and overall survival. However, as this analysis is based on statistics on a group of heterogeneous types of soft tissue/sarcomas with different genomic alterations and clinical behavior, a conceivable correlation for particular entities may be concealed. Additional studies with larger data sets are therefore needed to perform survival analysis for individual types of soft tissue/sarcoma to further validate the utility of YAP1/TAZ as prognostic biomarker.

Providing further evidence of the activated state of YAP1/TAZ in MLS, SySa and MPNST, nuclear YAP1/TAZ expression levels were demonstrated by immunoblotting of cytoplasmic and nuclear protein fractions in cell lines. Consistent with this finding, suppression of nuclear YAP1/TAZ-TEAD transcriptional activity employing the small molecule inhibitor verteporfin resulted in a significant reduction of sarcoma cell growth and viability *in vitro* and *in vivo*, indicating requirement for YAP1/TAZ activity and the potential of YAP1/TAZ-TEAD inhibition as a novel therapeutic approach in sarcoma patients with nuclear YAP1/TAZ activity.

Alterations in various effectors of the Hippo signaling cascade such as *YAP1, LATS2* or *SAV1* copy number variations have recently been described in a variety of sarcoma subtypes and point to a major role of aberrant Hippo signals in different soft tissue malignancies^6,30^. Seidel and colleagues demonstrated a frequent reduction of *MST1/2* and *LATS1* gene expression by promoter hypermethylation in soft tissue sarcomas^31^. In epithelioid hemangioendothelioma, aberrant Hippo signaling was shown to be induced through chromosomal translocations directly involving *YAP1* or *WWTR1* (encoding TAZ)^27–29^. In alveolar rhabdomyosarcoma, the chimeric PAX3-FOXO1 oncoprotein was found to promote tumorigenesis by inhibition of MST1, and dysregulation of YAP1^8^. We previously described an oncogenic mechanism of aberrant YAP1/Hippo signaling activation mechanistically based on the *FUS-DDIT3*^33^ and *SS18-SSX*^34^ oncoproteins in MLS and SySa which is to some extent reminiscent of the situation in alveolar rhabdomyosarcoma in which the pathognomonic PAX3-FOXO1 protein promotes tumorigenesis by Hippo pathway suppression^8^.

In conclusion, we analyzed the currently largest cohort of tumors of soft tissue and bone to identify strong nuclear expression levels as a common pattern of several sarcoma subtypes associated with a functional dependency on transcriptional YAP1/TAZ activity as specific liability in sarcoma cells. YAP1/TAZ signaling may therefore represent a target for therapeutic interventions in subgroups of tumors of soft tissue and bone which may be identified through immunohistochemical screening for nuclear YAP1/TAZ as biomarkers, to be prospectively addressed in future studies.

## Data Availability

The data that support the findings of this study are available on request from the corresponding author. The data are not publicly available due to privacy or ethical restrictions.
